# Mechanisms underlying pre- and postnatal development of the vomeronasal organ

**DOI:** 10.1007/s00018-021-03829-3

**Published:** 2021-04-19

**Authors:** Raghu Ram Katreddi, Paolo E. Forni

**Affiliations:** Department of Biological Sciences, Center for Neuroscience Research, The RNA Institute, University At Albany, State University of New York, Albany, NY USA

**Keywords:** Neuronal differentiation, Tfap2e/AP-2ε, Neurogenesis, Olfactory placode, Transcription factor

## Abstract

The vomeronasal organ (VNO) is sensory organ located in the ventral region of the nasal cavity in rodents. The VNO develops from the olfactory placode during the secondary invagination of olfactory pit. The embryonic vomeronasal structure appears as a neurogenic area where migratory neuronal populations like endocrine gonadotropin-releasing hormone-1 (GnRH-1) neurons form. Even though embryonic vomeronasal structures are conserved across most vertebrate species, many species including humans do not have a functional VNO after birth. The vomeronasal epithelium (VNE) of rodents is composed of two major types of vomeronasal sensory neurons (VSNs): (1) VSNs distributed in the apical VNE regions that express vomeronasal type-1 receptors (V1Rs) and the G protein subunit Gαi2, and (2) VSNs in the basal territories of the VNE that express vomeronasal type-2 receptors (V2Rs) and the G subunit Gαo. Recent studies identified a third subclass of Gαi2 and Gαo VSNs that express the formyl peptide receptor family. VSNs expressing V1Rs or V2Rs send their axons to distinct regions of the accessory olfactory bulb (AOB). Together, VNO and AOB form the accessory olfactory system (AOS), an olfactory subsystem that coordinates the social and sexual behaviors of many vertebrate species. In this review, we summarize our current understanding of cellular and molecular mechanisms that underlie VNO development. We also discuss open questions for study, which we suggest will further enhance our understanding of VNO morphogenesis at embryonic and postnatal stages.

## Introduction

The vomeronasal organ (VNO) is a specialized olfactory organ responsible for detecting pheromones and kairomones; stimuli that can trigger a wide range of behaviors [1–5] and hormonal responses [6–8]. Many vertebrates have a vomeronasal organ composed of a sensory and a nonsensory epithelium [9]; however, several adaptive/developmental differences can occur across species [10, 11]. Karl Von Baer’s principles state that the general anatomical features in animal species appear earlier in development than the more specialized features. In line with this, the vomeronasal anlage has been reported as a transient embryonic structure present in many vertebrate species, including birds [12, 13], where the VNO does not form, and humans, where a VNO-like structure has been documented only in rare cases [14–16]. This suggests that the embryonic vomeronasal anlage is a general structure that, depending on the animal species, may or may not develop into a functional VNO.

During the early development of the vomeronasal anlage of mice (E10.5–E11.5), some endocrine neurons, called gonadotropin-releasing hormone-1 (GnRH-1) neurons, form proximal to the prospective VNO and then migrate into brain. Once in the brain, these neurons become an integral part of the hypothalamic–pituitary–gonadal axis. GnRH-1 neurons release GnRH to trigger the release of gonadotropins, luteinizing hormone (LH) and follicle stimulating hormone (FSH), from the pituitary gland. These hormones are critical for proper gonadal function [17–20]. Recent 3D analysis of human embryos by Casoni and coworkers also found that GnRH-1 neurons form proximal to the embryonic vomeronasal structure and migrate to the brain in a process consistent with that in mice [21]. Based on these data, we posit that the embryonic vomeronasal anlage is an evolutionarily conserved structure.

During the late embryonic and adult stages, the mouse VNO is mostly composed of two main classes of vomeronasal sensory neurons (VSNs) that each selectively expresses G protein coupled receptors (GPCRs) encoded by one of the two vomeronasal receptor (VR) gene families: V1R and V2R [22–27]. Hundreds of VSN subpopulations expressing V1Rs or V2Rs bind different ligands, trigger distinct innate behaviors, localize in different regions in the VNO and project to different areas in the accessory olfactory bulb (AOB) [4, 28–32]. V1R positive neurons lie mostly in the apical zone of the vomeronasal epithelium and express the G-protein subunit Gαi2 and neuropilin-2 (Nrp-2) and project to the anterior portion of the AOB [33, 34]. V2R positive neurons lie in the basal zone of the epithelium and contain the G-protein subunit Gαo, the Slit receptor Robo2, and project to the posterior portion of the AOB [35]. A small subset of V2R and Gαo + VSNs co-expresses another multigene family of nine nonclassical class I major histocompatibility genes, H2-Mv genes. Gαo + VSNs that are positive for H2-Mv reside in the lower sub layers of the basal zone in the vomeronasal sensory epithelium [36–39].

Approximately a decade ago two independent research groups identified an additional subclass of Gαi2 or Gαo positive VSNs, which express formyl peptide receptors (FPRs) but do not overlap with V1R or V2Rs in rodents [40, 41]. 7 FPR genes exist in the mouse genome, though five were identified in the VNO. *Fpr-rs1* is co-expressed with the Gαo subunit in the basal zone, whereas *Fpr-rs3*, *Fpr-rs4*, *Fpr-rs6,* and *Fpr-rs7* are co-expressed with Gαi2 in the apical zone [40]. Later studies also revealed that Fpr-rs3 VSNs showed similar physiological properties to the remaining VSN population [42], and Fpr-rs1 VSNs have stereo-selective preference towards D-amino acid containing peptides, which specifically occur in pathogenic bacteria, viruses, and fungi [43].

Several studies refer to Gαi2 + VSNs as apical VSNs, while Gαo expressing VSNs are often named basal VSNs [44–47]. Even though the spatial distinction is not absolute, in this review, we will sometimes refer Gαi2 + and Gαo + VSN populations using the historically accepted apical and basal VSNs nomenclature, respectively.

In this review article, we will discuss some key aspects of embryonic and postnatal VNO development of murine vomeronasal organ, as excellent and extensive reviews exist on postnatal vomeronasal connectivity, adult neurogenesis and function [46–53]. Our goal is to highlight underexplored areas of developmental biology from our perspective.

## Ontogeny of the VNO

**Fig. 1:**
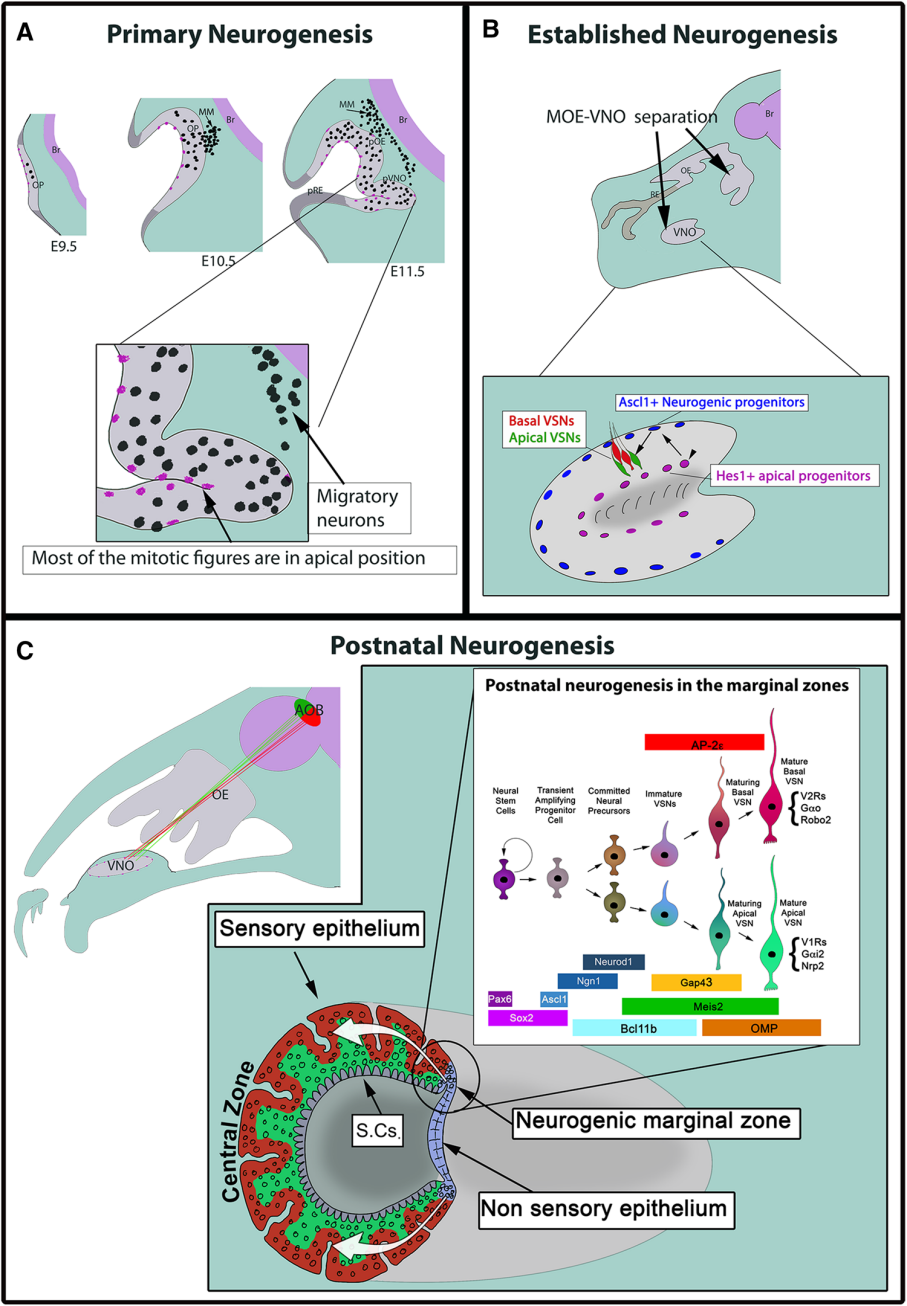
Schematic of VNO ontogeny in the sagittal view illustrating neurogenesis from embryonic to postnatal stages. **a** Schematic showing olfactory placode specification at E9.5, invagination of olfactory placode to form olfactory pit at E10.5 followed by vomeronasal thickening and invagination at E11.5. At this stage, most of the proliferative progenitors are localized to apical side of the epithelium (magenta dots). Between E10.5 and E11.5, multiple migratory neuronal populations (Black dots) leave the olfactory pit to form the migratory mass (MM). Neurons of the MM migrate from the olfactory pit towards the brain. **b** During established neurogenesis stage, VNO separates from the MOE. Inset shows proliferative apical progenitors, identified by Hes1 (magenta), and neurogenic basal progenitors positive for the transcription factor Ascl1 (blue). **c** Schematic showing Gαi2 + apical and Gαo + basal VSNs respectively sending their axons to the anterior (green) and posterior (red) portions of the AOB. Inset shows adult VNO in coronal view. Lumen separates the medial sensory epithelium and lateral nonsensory epithelium, with marginal zones in between them. In the vomeronasal sensory epithelium, basal VSNs are indicated in red, while apical VSNs are shown in green and sustentacular cell layer is labelled as S.Cs. Adult neurogenesis mostly occurs in the marginal zones (circled) of the VNO. The new born VSNs in the marginal zone migrate slowly towards the central zone of the epithelium (white arrows). A summary cartoon (white box) highlights the spatial and temporal specific expression of transcription factors during the formation and maturation of apical and basal VSNs starting from stem cells. Once the dichotomy is established, the transcription factor AP-2ε is selectively expressed by the basal neurons while Meis2 is restricted to the apical neurons. Additional apical and basal markers are listed close to the mature apical and basal VSNs. **a** Is based on [159, 160]; **b** is based on [27, 64] and **c** is based on [26, 76, 121, 126]

During embryonic development, both the olfactory and lens placodes share a common origin from the preplacodal ectoderm [54]. Over time, the spatial competence of the olfactory placode becomes restricted and specified to the most antero-lateral side of the head ectoderm [55]. In 1975, Cuschieri and Bannister first described the ontogeny of the mouse VNO [56]. The VNO arises from the olfactory placode, which is visible as a transient thickening in the antero-lateral region of the embryonic head at embryonic day (E) 9.5. Previous studies revealed two different phases of olfactory neurogenesis—early/primary neurogenesis and established neurogenesis (Fig. 1a, b) [57–59]. By E10–10.5, the olfactory placode invaginates to form the olfactory pit, which begins the early neurogenesis phase [58]. The olfactory pit is divided into anterior-medial sensory epithelium and posterior–lateral respiratory epithelium [60, 61]. By E11.5, the prospective vomeronasal organ thickens at the medial walls of the olfactory pit, which further invaginates toward the mesenchyme to form the vomeronasal groove [56].

In parallel with this stage, Tucker and colleagues identified two distinct populations of neural precursors that give rise to the major neuronal classes (eg. olfactory sensory neurons, VSNs and GnRH-1 neurons) in the nascent olfactory epithelium. These precursor cells are either (1) slowly proliferating, self-renewing precursors that express Meis1/2 at high levels that are localized primarily in the lateral olfactory epithelium or (2) rapidly proliferating, transit amplifying precursors that express high levels of Sox2 and Ascl1 and are localized primarily in the medial olfactory epithelium [62]. By E12.5, the vomeronasal groove on both sides of the embryo further extends as a tubular structure to form the primitive VNO that separates from the olfactory pit. The VNO, at this developmental stage, is distinguishable into sensory epithelia towards the medial side, and nonsensory epithelia toward the lateral side [56, 63]. As the developing VNO transitions into established neurogenesis, the VNO features three main cells types in the epithelium: (1) Hes-1 + apical proliferative progenitor cells that lie towards the lumen, (2) Ascl1 + basal neurogenic progenitors that arise from apical progenitors and settle towards the basement membrane, and (3) postmitotic VSNs in the intermediate zone [27, 56, 64–66] (Fig. 1b).

From E13.5, the VNO increases in size and length, such that a cross-section resembles a kidney shape with a crescent shaped lumen separating the sensory and nonsensory epithelia. Unlike the main olfactory epithelium (MOE), blood vessels and perivascular connective tissue in the vomeronasal sensory epithelium are detectable from embryonic to postnatal life [56, 67, 68]. The VNO opens anteriorly and connects to the nasal cavity through a narrow vomeronasal duct. The VNO is also composed of highly vascularized cavernous tissue with large blood vessel lateral to the nonsensory epithelium. The autonomic innervation to the blood vessels can cause vasoconstriction, which then can function as a vomeronasal pump to enable influx of external stimuli [69, 70]. Whether the murine VNO is functional at birth still remains unclear. While Coppola and colleagues showed that the vomeronasal duct is not open at birth [71, 72], others reported that the vomeronasal duct can open to external chemical stimuli at perinatal stages [73]. The development of the VNO is delayed as compared to the MOE, as VNO morphogenesis continues extensively in postnatal stages until sexual maturity [74]. After development is complete, the VNO continually generates new neurons throughout life [75]. BrdU-based birth dating studies at adult stages highlighted the presence of vomeronasal neurogenic progenitors at the marginal zone between sensory and nonsensory epithelia and at the central zone close to the basement membrane, which may mediate tissue homeostasis (Fig. 1c) [76–78].

## Early morphogenesis: early neurogenesis, GnRH-1 neuron formation, migratory mass and terminal nerve

Molecularly heterogenous populations of neuronal and non-neuronal cells arise from the olfactory placode as early as E10.5 in mice. These include non-neuronal cells of the putative respiratory epithelium, neuronal stem cells, olfactory sensory neurons, and various migratory neuronal cell types [79]. Migratory neurons formed during the early neurogenesis phase include cells immunoreactive for GnRH-1, Neuropeptide Y, TAG-1, olfactory marker protein (Omp), gamma amino butyric acid (GABA), tyrosine hydroxylase, and Islet-1 (Isl1) [18, 80–86] (Fig. 1a). Interestingly, most migratory cell populations are still poorly characterized both in terms of molecular markers and function. Some cell populations include neurons that are part of a complex ganglionic nerve called the terminal nerve [86] and “guidepost” neuronal cells that can aid in the initial formation of olfactory and vomeronasal nerve [79].

In mouse, the GnRH-1 neurons form in the olfactory placode between E10 and E11.5 proximal to the vomeronasal neurogenic area and start migrating by E11.5 from the nasal area to the forebrain [17, 18]. GnRH-1 neurons begin invading the brain around E12.5 and complete their migration to the basal forebrain around E16.5 [18]. Disturbances in GnRH-1 neuronal formation, migration or signaling negatively affect sexual development and fertility of mammals [17–21, 27, 87]. The phenotypic association between anosmia and defective GnRH-1 migration in Kallmann syndrome led to a prevailing model suggesting that migration of GnRH-1 neurons to the brain strictly depends on correct formation of the olfactory/vomeronasal system [19, 88–92]. However, other lines of evidence suggest that GnRH-1 neurons migrate to the brain along the axons of the *nervus terminalis* or terminal nerve (also cranial nerve-0) [17, 86]. The terminal nerve is still an ambiguous and controversial ganglionic nerve [93–96]. Some reports indicate that this nerve is a transient caudal branch of the vomeronasal nerve [97], while others suggest that the terminal nerve and vomeronasal nerve differ in neuronal composition, genetic expression, and guidance cue response [86]. In fact, Arx-1 null mice, a mouse model that lacks both OB formation and vomeronasal connections to the brain, still have terminal nerve projections to the hypothalamic area and successful GnRH-1 migration to the brain [86]. These data provided strong evidence that formation of the olfactory bulb and connectivity of vomeronasal and/or olfactory neurons to the olfactory bulb are not required for GnRH-1 neuronal migration into the brain. These data suggest that the broadly accepted causal relationship between defective development of the olfactory/vomeronasal system and aberrant GnRH-1 migration warrants revisiting [98]. Interestingly, a recent publication suggested that GnRH-1 neurons express some vomeronasal receptors [99]. These data suggest that the terminal nerve and GnRH-1 neurons, which are likely present in all vertebrates, could represent an ancestral form of the vomeronasal system. For more extensive reviews on GnRH-1 and terminal nerve please see [19, 94, 100–102].

The mechanisms underlying the formation of various early neuronal and non-neuronal sub populations in the developing olfactory pit remain unresolved. Whether the neurons formed during early and late neurogenesis in the developing olfactory pit have a common genetic lineage also remains a matter of debate. Studies in fish and chick identified the Isl1 transcription factor as a molecular marker for specific subpopulations of cells of the migratory mass (MM). In zebrafish, Isl1 expression colocalize with Gnrh3 neurons, whereas Isl1 + cells in chick were found as early migratory neuronal cells, distinct from Lhx2 + and GnRH-1 + neurons of the terminal nerve ganglion [103, 104]. Performing Isl1Cre genetic lineage tracing in mice, we found Isl1Cre recombination/lineage in almost all GnRH-1 neurons, cells of the migratory mass, cells of the respiratory epithelium, in sparse olfactory, vomeronasal neurons and supporting cells [84]. However, Isl1 conditional knockout from GnRH-1 neurons in mice did not cause any disturbance in their formation or migration, leaving the role of this transcription factor in GnRH-1 neurons and other cells formed during the early placodal neurogenesis unresolved [84].

## Intrinsic and extrinsic factors regulate VNO development

### Transcriptional regulation during early development

As the VNO is formed after a secondary invagination from olfactory pit (Fig. 1a, b), all the transcription factors and signaling molecules that affect the formation of the olfactory placode/pit may also control VNO development (Table 1). In this section, we will highlight several studies that showed disruption of olfactory placode/pit and VNO development due to mutation of different transcription factors during the primary neurogenesis phase.

**Table 1:** Transcription factors playing a role in olfactory/VNO development

Transcription factor	Phenotype	References
Pax6	No olfactory placode	Grindley et al. [105]
Sox2	MOE disrupted by E10.5 VNO phenotype not described	Panaliappan et al. [60]
Six1	No VNO or MOE formation	Ikeda et al. [57]
Six1; Six4	No olfactory pit	Chen et al. [108]
Foxg1	No VNO formation	Duggan et al. [111]
Dlx5	VNO rudimentary or lost	Long et al. [114]
Fezf2	No VSNs by P0	Eckler et al. [115]
Ascl1	Drastic reduction in VSNs	Murray et al. [121]
Ascl1; Ngn1	Reduction in VSNs	Cau et al. [66]
N-myc	Reduction in proliferation and VSN neurogenesis	Wittmann et al. [59]
Gli3	Reduction in Ascl1 + cells and VSNs	Taroc et al. [27]
Bcl11b	Increased Gαi2 + VSNs and decreased Gαo VSNs	Enomoto et al. [26]
Tfap2e/AP-2ε	Reduction in basal VSNs and change in basal VSN identity	Lin et al. [126]
Atf5	Reduction in basal VSNs	Nakano et al. [127]

Pax6, a member of the Pax transcription family, is expressed in both olfactory and lens placodes during early development. Grindley and coworkers [105] used Pax6 homozygous mutant (*Sey*) mice to study its role in placodal formation. In these mutants, they found no lens, olfactory placode thickening, nasal cavity, and olfactory bulb formation. Subsequent studies using chimeric embryos composed of both wildtype and Sey (Pax6^−/−^) mutant cells [106, 107] showed that only wild type cells, not Pax6 mutant cells, participate in olfactory placode invagination. These data confirmed a cell autonomous role for Pax6 in olfactory placode formation.

Six1 and Six4 transcription factors, members of Six gene family, are expressed in the preplacodal region even before the specification of the olfactory placode. Two studies by Ikeda and coworkers reported a shallow olfactory pit invagination in Six1^−/−^ mice as compared to the wild type mice at E10.5 [57, 58] and showed that Six1 is critical for the differentiation of stem cell progenitors into neuronal precursors. In fact, in Six1^−/−^ mice the VNO does not form by E12.5, and the olfactory epithelium degenerates by E14.5. Using a Six1; Six4 double knockout mouse model, Chen et al. [108] showed synergistic requirements of both Six1 and Six4 for olfactory placode and VNO development. In these double knockout mice, the olfactory placode does not invaginate to form an olfactory pit. Notably, no defects were observed in the olfactory or VNO development in the Six4^−/−^ mice alone, highlighting the functional redundancy of the Six family transcription factors [109].

Sox2, a SoxB1 family member, is also expressed from the early olfactory placode stage and may serve as a stem cell marker of both the olfactory and vomeronasal epithelium [63]. Panaliappan and coworkers reported that conditional ablation of Sox2 disrupts the olfactory epithelium by E10.5 due to increased apoptosis, reduced proliferation and diminished neurogenesis [60]. Sox2 is essential in promoting neurogenic lineage by restricting Bmp4 expression and regulating Hes5 expression in the nasal epithelium [60, 61]. In addition, another study showed the ability of Sox2 to bind to the enhancer elements and induce the expression of Pax6, one of the initial olfactory placodal markers [110].

Foxg1, a Forkhead family transcription factor, is another transcription factor expressed throughout the developing olfactory placode. Duggan and coworkers placed Foxg1 expression upstream to stem cell progenitor marker Ascl1, in the proneural genes cascade that controls both olfactory and vomeronasal neurogenesis [111]. Moreover, studies on Foxg1 constitutive knockout mice showed reduced proliferation and increased apoptosis at the early placodal development [112] and lack of VNO formation.

Dlx5 is a homeobox transcription factor that contributes to the development of the olfactory placode and olfactory bulb in mice [113, 114]. Dlx5 mutant mice display a right-left asymmetry in the nasal cavity formation together with complete absence or rudimentary development of the VNO. Dlx5^−/−^ mutants fail to produce olfactory or vomeronasal projections to the main and accessory olfactory bulbs.

All these transcription factors are expressed in the olfactory placode stage, so their mutations also affect VNO formation. However, spatially distinct inductive signals and transcriptional factor networks may be quite critical for the vomeronasal thickening or invagination between E10.5 and E11.5. Nevertheless, Fezf2 remained the only known transcription factor, to our knowledge, that mechanistically contributes to VNO morphogenesis. Both Fezf1 and Fezf2 are closely related zinc finger transcription factors with 97% percent similarity in their zinc finger moiety [115]. Hirata and colleagues showed that Fezf2/Fezfl, is specifically expressed in the VNO at E12.5 [116], while Fezf1 is highly expressed in the MOE and weakly expressed in the VNO [117]. Then, Eckler and colleagues reported that Fezf2 expression is present as early as E10.5 at the vomeronasal thickening of the olfactory pit, which makes it one of the earliest markers for the vomeronasal area [115]. As age increases postnatally, Fezf2 expression becomes restricted to the sustentacular cells. Even though Fezf2−/− mice show normal separation of the VNO from the olfactory pit by E13.5, the VNO degenerates by the date of birth in these mutants. Fezf1−/−; Fezf2−/− double mutants did not reveal obvious synergistic effects in the VNO, but did show increased expression of vomeronasal specific genes in the MOE [115]. This study proposed that both Fezf1 and Fezf2 repress the expression of VSN-related genes in the main olfactory and vomeronasal epithelium, respectively. Nevertheless, the role of Fezf2 in the progenitor cells during early development of the VNO and the mechanisms of neuronal cell death in these knockout mice still remain unknown.

### Transcriptional regulation of VNO development during established neurogenesis

After the invagination of the vomeronasal pouch occurs, migratory neuronal populations leave the VNO toward the brain, and the neurogenesis of VSNs begins (Fig. 1a) [27, 84, 118]. During the established neurogenesis phase, the VNO differentiates into medial sensory and lateral nonsensory epithelium with the lumen separating them [56, 63]. Even though only a few neurons form on the nonsensory side, they tend to undergo cell death as age increases [119, 120]. As the stem cells proliferate and differentiate towards VSNs, transcription factors express dynamically at specific temporal stages and play a key role in the VSN development (Table 1).

Ascl1, Ngn1, and Neurod1 are a neurogenic basic-helix-loop-helix (bHLH) family of transcriptional factors that are expressed during VSN development in the same temporal sequence as the MOE [121, 122]. These factors are essential for the specification and differentiation of the neurogenic progenitors. In the VNO, Ascl1 is predominantly expressed by basal progenitors, which further divide and give rise to immediate neuronal precursors that consequently express Ngn1 and Neurod1 [27, 121]. These Neurod1 + precursors mature into the two major types of vomeronasal neurons—Gαi2+ apical and Gαo + basal VSNs in the VNO [26]. Ascl1 knockout mice showed drastic reductions in both apical and basal VSN populations highlighting the role of Ascl1 in VSN development [27, 121]. Notably, though very few in number, VSNs can form in Ascl1/Ngn1 double KO mice, suggesting the existence of redundant or compensatory neurogenic factors in the VNO [66].

During embryonic development, the transcriptional regulator Gli3 is expressed along with Hes1 in the apical progenitors in the VNO [27]. Gli3 acts as transcriptional repressor in the absence of Shh signaling, while Hes1 is a bHLH transcriptional repressor that is essential for repressing neurogenic factors (e.g., Ascl1, Ngn1, Neurod1) [64]. In the VNO, Gli3 showed a similar role to the one described in the cortex in controlling the transitions of stem cells from proliferative to neurogenic program [27, 123]. In fact, characterization of Gli3 null mutants showed a drastic reduction of vomeronasal Ascl1 + neurogenic progenitors and VSNs [27].

The expression of the bHLH transcription factors Hes5 and Hes6 has also been documented in the vomeronasal sensory epithelium; however, loss of function studies are not conducted till now to study their role in VNO development [59, 64, 124].

N-myc is one of the myc proto-oncogene family members that is expressed as early as E9.5 in the olfactory placode at the onset of neurogenesis. Wittmann and colleagues studied the role of N-myc in olfactory and vomeronasal development using N-myc^Foxg1Cre^ conditional knockout mice [59]. N-myc deletion caused a reduction in the proliferation and neurogenesis, with a complete loss of Hes5 positive progenitors in both olfactory and vomeronasal epithelia specifically during established neurogenesis. Furthermore, mutant mice also showed reduced neuronal cell size, ultimately leading to severe atrophy of VNO and olfactory epithelium.

#### Dichotomy and maturation of apical and basal VSNs

Both the MOE and VNE originate from the common olfactory primordium and utilize similar transcriptional cascades to control neuronal specification and differentiation [66]. However, neuronal cell type diversity is one of many prominent features that distinguish VNO from MOE. The VNO contains Gαi2 + apical and Gαo + basal VSNs as the two major neuronal types that are spatially segregated with respect to the basement membrane (Fig. 1c). Yet, the mechanisms that underlie cell fate determination is not fully understood. Bcl11b remains one of the earliest identified transcriptional factors that contributes to Gαi2 vs Gαo + VSN cell fate determination [26]. Bcl11b expression begins at the vomeronasal groove stage by E11.5 [26]. Newborn Bcl11b–/– mice have a dramatic decrease in mature VSNs due to increased apoptosis. These mutant mice also display a reduction in the number of Gαo+ basal VSNs that selectively express the Tfap2e/AP-2ε transcription factor and an increased proportion of Gαi2 + apical VSNs that selectively express the transcription factor Meis2.

These data highlighted the contributions of Bcl11b in the fate choice between Gαi2 + apical vs Gαo + basal VSNs [125]. Later genetic lineage tracing confirmed that the transcription factor tfap2e/AP-2ε is expressed specifically in Gαo + basal VSN cell lineage [126]. Moreover, characterization of AP-2ε null mice showed that the loss of AP-2ε negatively affects the basal neuronal differentiation/maturation program [126]. In particular, loss of AP-2ε function induced a progressive loss of basal VSNs and aberrant gene regulation. In AP-2ε null mice, many cells that entered the basal program gradually expressed apical genes, such as Gαi2, V1Rs and Meis2. Based on these results, we proposed that AP-2ε is not needed to initiate the apical–basal VSN differentiation dichotomy, but instead is essential to maintain the basal VSN program and prevent the expression of apical VSN genes [126]. What role AP-2ε plays in controlling the chromatin landscape and expression of basal VSN specific genes requires further investigation.

Activating transcription factor 5 (ATF5) is another transcription factor that participates in the maturation of basal specific VSNs [127]. ATF5 is a member of the ATF/cAMP response element-binding (CREB) family of transcription factors and has well-established prosurvival activity in different organs, including the MOE [128]. ATF5–/– mice showed a dramatic reduction in OMP positive mature VSNs specifically in the basal neuronal lineage due to increased apoptosis [127]. A follow-up study suggested that ATF5 may form a heterodimer with CCAT/enhancer binding protein gamma transcription factor, which then enhances the transcription of vomeronasal receptors [129]. However, the reasons for the specific reduction of Gαo+ basal VSNs, not Gαi2 + apical VSNs, in ATF5 knockout mice are still unknown.

A recent study by Chang and Parrilla [130] further characterized the expression of 28 homeodomain transcription factors in different postnatal VNO cell populations including neuronal progenitors, precursors, neurons and non-neuronal cells. However, specific roles of these proteins in VNO development and maturation require further investigation. In addition to these transcription factors, proteins that comprise a vomeronasal signaling cascade are also vital for the maturation, survival and functionality of the vomeronasal neuronal network in the VNO [4, 28–31, 131–133].

### miRNAs in vomeronasal neurogenesis

Transcription factors are only a part of a complex regulatory system that aid neurogenesis. For example, microRNAs are a major class of noncoding RNAs that regulate the expression of transcription factors. In the MOE, conditional knockout of Dicer complex, an enzyme required for miRNA production, at the neuronal progenitor stage (Foxg1Cre) showed a degeneration of olfactory neuroepithelium [134]. In these mutants, vomeronasal thickening and invagination did not appear to be perturbed at E11.5; however, this study did not report VNO development in the later stages. Notably, Omp Cre mediated Dicer ablation at a mature neuronal stage did not affect the number of olfactory/vomeronasal neurons, axonal guidance, glomeruli formation or animal behavior. This further underscore the importance of miRNAs during the early stages of neuronal proliferation and differentiation in the olfactory system. A separate study specifically analyzed the role of Dlx5 on *miR-9* and *miR-200* class miRNAs expression and how they affect the differentiation of olfactory/vomeronasal neurons [135]. Dlx5–/– mutant mice showed a downregulation of *miR-9* and *miR-200* class microRNAs and an upregulation of Foxg1 protein in the olfactory epithelium. Moreover, this study confirmed that these microRNAs can target the 3′UTR of the Foxg1 mRNA to inhibit its translation. This study not only connects the Dlx5 and Foxg1 regulation in the OE and VNO development, but also highlights how noncoding RNAs can participate in a complex transcriptional regulatory network controlling neuronal development.

### Inductive signals in VNO development

**Fig. 2:**
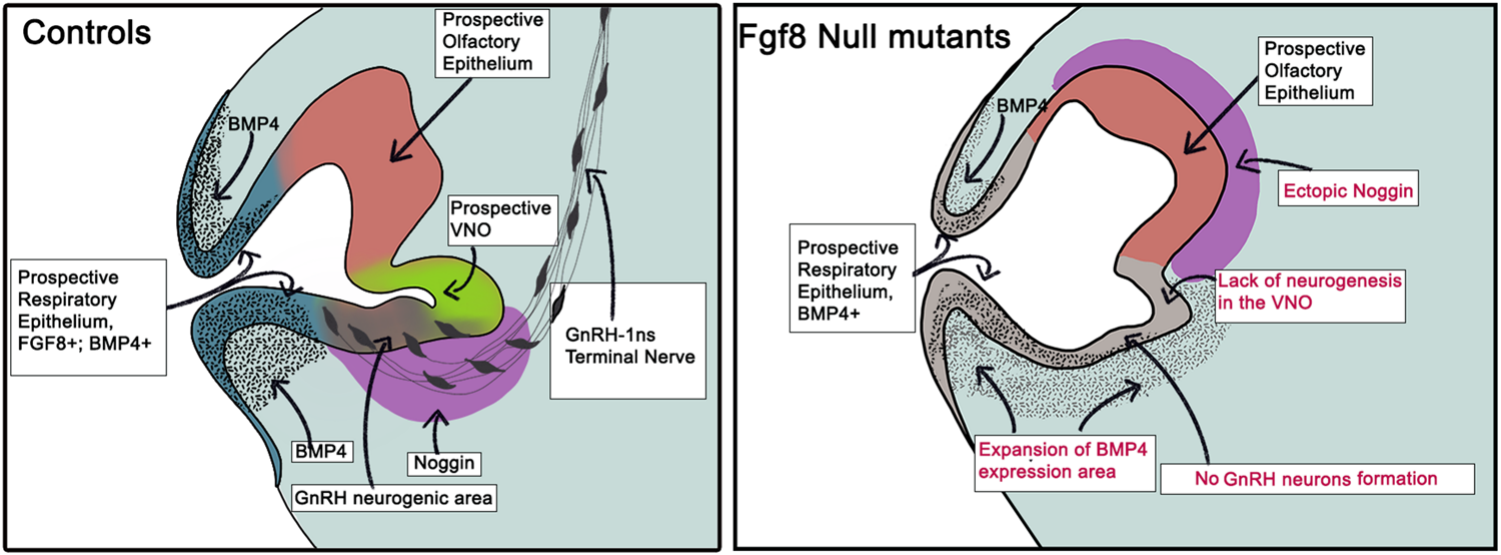
Cartoon summarizing some changes observed in FGF8 null mutants. Fgf8 (blue) is expressed by the cells forming the presumptive respiratory epithelium. Bmp4 (black dotted pattern) is also expressed in respiratory epithelium and by underlying mesenchyme, which further regulate the expression of Bmp4 antagonist Noggin (magenta) close to the GnRH-1 and vomeronasal neurogenic area. The expression of mesenchymal Noggin correlates with the formation of GnRH-1 and vomeronasal neurons. In Fgf-8 null mutants, Bmp4 expression expanded into the vomeronasal and mesenchymal area disrupting the noggin source. Fgf8 mutants do not form GnRH-1 neurons and have no vomeronasal neurogenesis. Figure 2 is based on [118, 154]

**Fig. 3:**
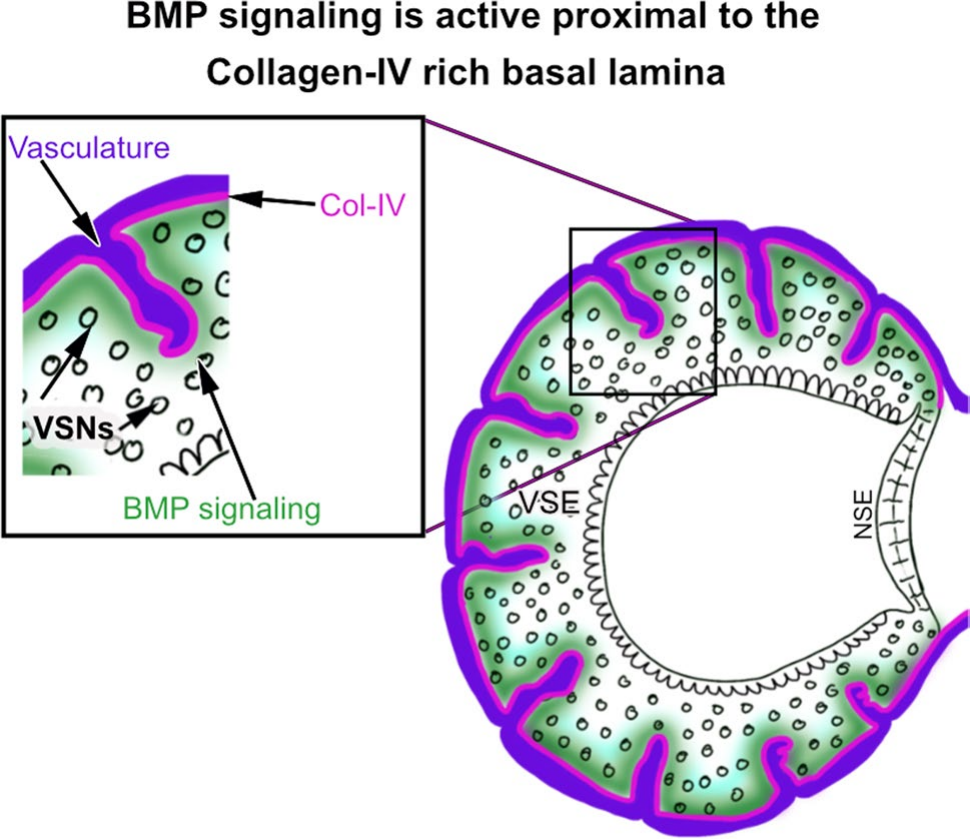
Schematic illustrating the relation between Collagen IV (Col-IV) positive basement membrane (magenta) and active BMP signaling (green) in the basal VSNs in the adult coronal VNO section. Collagen IV invades the basal regions of the VNO surrounding the vasculature (purple). The basal lamina can aid in sequestering the Bmp that generates Bmp signaling gradients in the basal territories of the VNO with stronger Smad1,5,8, activation (green) close to the basal lamina. VSNs- Vomeronasal sensory neurons; VSE- Vomeronasal sensory epithelium; NSE- Nonsensory epithelium Fig. 3 is based on [68]

Extracellular cell signaling pathways also play an important role in olfactory placode determination and subsequent differentiation into respiratory, olfactory and vomeronasal epithelia. Inductive signals within the ectodermal cells or between the ectoderm and underlying mesenchyme are essential to induce the spatial and temporal changes in the ectodermal gene expression that are ultimately required for olfactory placodal patterning [54, 136, 137]. For example, culturing olfactory placodal ectoderm in vitro*,* after separating it from the underlying mesenchyme before morphogenesis, prevented the specification of olfactory epithelium [138]. However, recombining and culturing the ectoderm and mesenchyme led to distinct ectodermal thickening, invagination resembling in vivo olfactory pit formation and subsequent sensory neuron differentiation. This experiment highlighted the importance of epithelial and mesenchymal interactions to induce specific cell fate decisions during the development of olfactory and vomeronasal epithelia. Indeed, studies reported the presence of both juxtacrine and paracrine inductive signals that interact to trigger the expression of specific transcription factors in the epithelia [139]. In this section, we will briefly discuss a few studies that highlighted the role of different signaling pathways underlying VNO development during both early and established phases of neurogenesis (Figs. 2, 3).

Fibroblast growth factors (FGFs) are polypeptide growth factors that participate in cell proliferation, differentiation, and organogenesis. Multiple factors like Fgf3, -8, -9, -10, -15, -17 and -18 are expressed near the nasal pit region [140]. Of the various Fgfs, the role of Fgf8 has been studied in more detail. Initial studies showed Fgf8 expression at the rim of the invaginating olfactory pit between E10 and E11 and proposed the presence of primordial neural stem cells of the olfactory epithelium positive for both Sox2 and Fgf8 [118, 141]. Fgf8 loss of function induced the failure of both MOE and VNO development due to increased apoptosis [141]. In a subsequent study in chick and mice, Maier and coworkers [61] showed that Bmp and Fgf signals specify respiratory and sensory epithelial cell fates, respectively. This study showed that Fgf8 activity restricts Bmp signals to respiratory epithelium, aiding the expression of neuronal genes in the sensory epithelia. However, Forni and coworkers [118] demonstrated a complex relationship between Fgf8 and Bmp4 signaling pathways with contributions from the underlying mesenchyme as an additional factor in respiratory vs neuronal specification (Fig. 2a). The authors did not find Fgf8 expression in Sox2 or Pax6 positive neural stem cells. Using Fgf8Cre lineage tracing, they suggested that cells positive for the Fgf8 lineage were restricted to the respiratory epithelium. They also reported Bmp4 expression both in Fgf8 positive respiratory ectoderm along with the underlying mesenchyme. In response to BMP4, strong expression of noggin, a Bmp4 antagonist, was found in a distinct group of mesenchymal cells proximal to the developing VNO. This Noggin source may facilitate neurogenesis in the sensory epithelium, thus delineating respiratory vs neurogenic domains. The authors also proposed that the effect of Fgf8 inactivation on olfactory and VNO disruption is indirect and due to a broader Bmp4 expression, which expanded into the VNO region preventing neurogenesis (Fig. 2b). A separate study probed the role of Fgf3 and Fgf10 in VNO development using Fgf3–/– and Fgf10–/– single mutants and double Fgf3–/–; Fgf10–/– mutation. However, no morphological or functional differences were identified in these mutants [142].

#### Do inductive signals aide in defining neuronal identity in postnatal life?

The mammalian main olfactory and vomeronasal epithelia display continuous neurogenesis throughout adulthood. However, our knowledge is limited regarding the role of inductive signals that control cell patterning, neuronal differentiation, and neuronal homeostasis in postnatal life. Basal/Gαo + VSNs that express different V2R receptors appear distributed in specific sublayers of the basal territory of the VNO [38]. Moreover, another study on the MOE showed that the spatial position of stem cells in the MOE can determine the identity of olfactory neuronal subtype [143]. These data suggest that local inductive signals in the main and accessory olfactory systems could play a role in defining the identity of neurons based on their relative positions in the epithelia.

Functionality of the accessory olfactory system relies on the correct development of VSNs, the ability to detect and transduce signals, and the establishment of the correct glomerular map in the AOB [4, 28, 46, 132, 144–147]. Our lab recently reported that active BMP intracellular signaling (phospho Smad1,5,8) is mostly restricted to VSNs in the basal regions of the VNO. Notably, the basal lamina of the vomeronasal epithelium, and the vasculature penetrating the VNE, is a rich source of Collagen-IV (Col-IV), a molecule with high affinity for BMP4 (Fig. 3) [148]. By using AP-2εCre mice to conditionally knock out Smad4 in maturing basal vomeronasal neurons, we found that Smad4 mediated TGF/Bmp signaling is important for proper dendritic knob formation, pheromone-induced VSN activation, survival, and correct glomerular formation of Gαo + basal VSNs in the posterior AOB. However, when we used OmpCre driver to knock out Smad4 from both apical and basal VSNs at a mature stage, we only found glomerular connectivity defects in the posterior AOB that reflected basal VSNs projections. These data suggest that morphogenic inductive signals in postnatal stages are also critical for proper basal VSNs development and connectivity to the posterior AOB [68].

## Conclusion and open questions

Owing to its vestigial nature or lack of function in adult humans, investigation of VNO development and function is limited to a small number of labs (grantome.com). Regardless of the formation of a functional postnatal VNO, the presence of embryonic vomeronasal anlage that gives rise to migratory cell populations, such as the GnRH-1 neurons, cells of the migratory mass and the terminal nerve (Fig. 1a), does occur in many animal species [17, 18, 21]. Perturbations affecting the formation or migration of early migratory cells can lead to reproductive disorders like isolated forms of hypogonadotropic hypogonadism with a normal sense of smell and those associated with a lack of sense of smell (Kallmann syndrome) [27, 149–152]. Understanding the mechanisms that lead to the formation and migration of multiple neuronal and non-neuronal populations originating from olfactory placode will exponentially advance our knowledge to discover novel therapeutic strategies for these disorders. In addition, the VNO of rodents is an excellent model system to study neurogenesis, cell fate determination, axon guidance, and behavior [26, 34, 35, 144, 145, 153]. In this review, we highlighted some transcriptional factors, miRNAs, and inductive signals that are important for the VNO development.

Until recently, only a few studies explored the molecular mechanisms underlying vomeronasal invagination and segregation from a common olfactory primordium. Fezf2 is shown to have VNO specific expression at the vomeronasal thickening stage during early development [115]. However, in Fezf2–/– knockout mice, initial VNO formation is observable until E13.5, which further suggests that it is not an essential factor in defining and segregating the VNO during the primary neurogenesis stage. In addition, Fgf8 can indirectly influence vomeronasal establishment via Bmp-induced noggin expression in the mesenchyme [118, 154]. We suggest that future investigations into the role of additional intrinsic and extrinsic factors both in the ectoderm and underlying mesenchyme may reveal additional mechanisms involved in the VNO formation. During the established phase of neurogenesis, Bcl11b is critical for the Gαi2 + apical vs Gαo + basal VSN cell fate determination [26]. However, this view of neuronal cell fate determination seems rather simplistic. Further studies should identify the mechanisms that induce Bcl11b expression and subsequent apical vs basal VSNs’ fate determination.

In the MOE, studies identified signaling pathways that provide key feedback for regulating olfactory neurogenesis [155]; however, such mechanisms remain unknown in the VNO. One interesting question is to study whether there is one common or distinct negative feedback mechanisms to control neurogenesis of Gαi2 + apical and Gαo + basal VSNs. Further outstanding questions remain—what signaling mechanisms are involved in the separation of the VNO into sensory and nonsensory epithelium during VNO development and why adult neurogenesis still occurs at the marginal zones of the VNO. Studying the role of cell autonomous factors vs those surrounding the stem cell niche in the adult neurogenesis at marginal zone of the postnatal VNO is a worthwhile endeavor.

Moreover, the role of Sustentacular cells has primarily been studied in the MOE. Few studies highlighted differential expression of metabolizing enzymes and genes in the sustentacular cells of the MOE as compared to the VNO [121, 156]. Do sustentacular cells in the MOE and VNO have similar functions? Furthermore, factors that induce specification of neuronal vs sustentacular cells from a common multipotent stem cells are still unknown in the VNO. Interestingly, the recent studies from the MOE using postinjury regeneration model found a role of Wnt and Notch signaling pathways in differentiating stem cells towards neuronal and non-neuronal supporting cells respectively [157, 158].

In conclusion, many open questions still remain about the formation of early migratory neuronal populations and the specification of the embryonic vomeronasal structure. Moreover, the key molecular mechanisms controlling neurogenesis and cell differentiation in the postnatal vomeronasal organ are still unresolved. The embryonic VNO anlage likely gives rise to migratory cells that control sexual development, while the postnatal VNO plays a crucial role in social interactions of animals. Further understanding of the molecular mechanisms underlying the development of the pre- and postnatal vomeronasal organ will impact the fields of neural development, evolutionary biology, ethology, and the medical field.
